# Internal consistency of the readiness for interprofessional learning scale in German health care students and professionals

**DOI:** 10.1186/1472-6920-14-145

**Published:** 2014-07-16

**Authors:** Cornelia Mahler, Justine Rochon, Sven Karstens, Joachim Szecsenyi, Katja Hermann

**Affiliations:** 1 Department of General Practice and Health Services Research, University Hospital Heidelberg, Vossstrasse 2, 69115 Heidelberg, Germany; 2 Institute of Medical Biometry and Informatics, University of Heidelberg, Heidelberg, Germany

**Keywords:** Interprofessional learning, Internal consistency, Questionnaires, Readiness for interprofessional learning, Assessment

## Abstract

**Background:**

The implementation of a bachelor degree in “Interprofessional Health Care” at the University of Heidelberg, Germany has fostered the need to evaluate the impact of this innovative programme. The Readiness for Interprofessional Learning Scale (RIPLS) was developed by Parsell and Bligh (1999) to assess student’s attitudes towards interprofessional education. The RIPLS consists of 19 items and four subscales were identified by McFadyen (*J Interprof Care***19:**595–603, 2005): “teamwork and collaboration”, “negative professional identity”, “positive professional identity” and “roles and responsibilities”. The RIPLS has been translated into a number of languages and used in a variety of different educational settings. A German version of the RIPLS was not available. Aim of the study was the translation of the RIPLS into German and testing of internal consistency.

**Methods:**

The RIPLS was translated to German according to international guidelines and its psychometric properties were assessed in two online surveys with two different samples a) health care graduates and b) health care students. Descriptive analysis (mean, SD, corrected item-total correlation) of the Readiness for Interprofessional Learning Scale – German (RIPLS-D) was performed for item characteristics and Cronbach’s Alpha was calculated for internal consistency of overall and subscales of the RIPLS-D.

**Results:**

Each sample consisted of 76 datasets. Reliability for the RIPLS-D overall scale was 0.83 in both samples. The subscales displayed internal consistency between 0.42 and 0.88. Corrected item-total correlation showed low values in two subscales in the sample of graduates.

**Conclusions:**

While the overall RIPLS-D scale is reliable, several subscales showed low values and should be used with caution to measure readiness for interprofessional learning in the German health care context. Internal consistency of the instrument does not seem to be given in health care professionals at different stages of their professional career. In particular the sub-scale “roles and responsibilities” was problematic. For these reasons, the RIPLS-D cannot be recommended for use to assess this concept.

## Background

Effective collaboration between health professionals improves patient care outcomes and increases patient satisfaction [1]. However, the 2010 Lancet report states that health care students today are not being adequately prepared for interprofessional collaboration due to the siloed nature characterizing most health professions education and socialisation [2]. Emerging policies and interprofessional frameworks such as that from the World Health Organisation are encouraging educational institutions to bring interprofessional learning into curricula [1,3].

Introducing interprofessional learning requires rigorous evaluation in order to assess impact and build evidence, for example, as to whether through such experiences students actually acquire interprofessional competencies [4]. For evaluation of interprofessional education, the modified Kirkpatrick framework has been suggested by Freeth et al. [4], which can assess impact of interprofessional learning on students. Longitudinal data of healthcare professionals at different educational stages is also vital as attitudes, expectations and beliefs of students related to interprofessional learning are assumed to change and develop over time [5].

The Readiness for Interprofessional Learning Scale (RIPLS) is one of the most frequently applied instruments for evaluation of interprofessional education and learning activities [6,7]. It has been used in different English speaking countries (UK [8,9], US [10,11], Canada [12,13], Australia [14-17], New Zealand [18]), different settings (undergraduate [18-20], postgraduate [21], non-Western [22]) and among a wide range of health professions (for example: physicians, nurses, physiotherapists, occupational therapists, speech therapists, physician assistants, dietetics, pharmacists, dentists, social workers). It was developed in 1999 by Parsell and Bligh based on evidence from the literature and expert panel input. RIPLS is a self-report instrument to assess the readiness of students to engage interactively with students of other health professions in learning together. RIPLS consists of 19 items in three subscales labelled “teamwork and collaboration”, “professional identity” and “roles and responsibilities”. The items are measured on a five-point Likert scale (1 = strongly disagree, 2 = disagree, 3 = undecided, 4 = agree, and 5 = strongly agree).

McFadyen et al. proposed a more stable subscale model consisting of four scales in which the original subscale “professional identity” was divided into two subscales labelling them “negative professional identity” and “positive professional identity” [23]. McFadyen’s two cohorts consisted of undergraduate students coming from seven health and a social care programme, one cohort being the control group and the other having being exposed to IPL activities. RIPLS has been translated into Swedish [24] and Japanese [25] showing good psychometric properties in the teamwork and collaboration subscale with the need for further refinement and development in the other scales. French Canadian [26] and Serbian [27] translations are also available. The aim of this paper is to describe the translation process of RIPLS into German, to show internal consistency of the translated German instrument and to discuss the use of this instrument at different education stages to measure longitudinal changes.

## Methods

### Translation process

The original version of the RIPLS was translated and adapted for use in Germany, according to international guidelines [28,29]. Permission from the developer of an instrument is required for cross-cultural translation, as well as appraisal of the back translation to ensure conceptual equivalence. As no contact to the original developer of the instrument, Parsell, was possible, developer colleagues (Mattick and Bligh) gave permission for translation to German, as to their knowledge a German translation was not available. However, the appraisal of a back translated German version by them was not possible. Therefore, translation was performed according to the two panel approach by Swaine-Verdier et al. [30], which does not involve a back translation [30]. This approach consists of two panels, one for translation from the original language to the new language and one panel for revision and review of the first version. Dual translation panels with lay people have been successful in producing conceptual equivalent quality of life instruments [30]. This approach was developed as no evidence supports that the back translation process is superior to alternative methods [31].

The RIPLS was translated independently from English to German by two people with a health care background (Physiotherapist [SK] and Registered Nurse [CM]). Both are German native speakers with excellent English language skills. Both versions were reconciled and subsequently synthesized into one final version. During the reconciliation process questions arose in the conceptualization of individual items. For example: what was the difference between the terms “students”, “health care students” and “other health care students”? and “In which items do they refer to students of other health care disciplines, when to students in general, and when to students of the same discipline?”. Items 5 and 8 refer to “skills” that the students attain. The translators were not quite sure whether they refer merely to “skills” or rather “competencies”. These questions were forwarded to the developers, however, they could not be readily resolved. The translators had to therefore come to independent agreement on wording of the items in a process of reasoning out conceptual understandings. The translated version was then piloted with a panel of seven German health care professional educators (two general nurses, a paediatric nurse, a speech therapist, a physiotherapist, a midwife and a radiographer). This led to rewording and grammatical adaptation of items 15 and 18 to increase readability and understanding in the German version, while not changing the original meaning. At the end of this process, a final version was established.

### Participants

The German RIPLS version (RIPLS-D) was administered in two online-surveys to a) heath care students in their first year of training and b) health care professionals approximately one year after graduation. Between August and November 2011, an email with the link to the online survey was sent to 267 students in their first year of training and 225 graduates. The educational institutions they had or were attending were either the Academy of Health Professions of the University Hospital Heidelberg or the Willy Hellpach Schule (health care vocational training school) also in Heidelberg. The health care professions represented included: geriatric nursing, paediatric nursing, general nursing, speech therapy, physiotherapy, midwifery, orthoptics, medical laboratory assistants, medical radiology assistants, health care assistants. A letter providing the online link to the survey was also sent to the postal home address of graduates whose email bounced back or who had no email address. A reminder was sent 2 weeks later. Besides RIPLS, the survey also included other scales measuring work satisfaction, research utilization, self-efficacy as well as socio-demographic data. Neither sample group had experienced interprofessional learning to the best of our knowledge. The RIPLS-D was sent to two cohorts of health professionals (students and graduates) at different stages of professional development to enable the research group to evaluate internal consistency of the instrument in each cohort. In order to compare data at different stages with a single instrument, internal consistency of an instrument is required.

This research was approved by the Ethics Committee of the Faculty of Medicine Heidelberg (graduates: S-239/2011; students: S-430/2011). In addition, permission was gained from the University Hospital Heidelberg as nursing students participants were employed there during vocational training.

### Statistics

Descriptive statistical analysis was carried out to assess participant characteristics as well as item and scale characteristics of the RIPLS in each sample. RIPLS-D scale and subscales were summarized by using means and standard deviations (SD), categorical data by using frequency counts and percentages.

Items 10 to 12 were reverse scored as suggested by McFadyen) [23]. Cronbach’s alpha was used to determine internal consistency of the total scale and the original 3 subscale model (Parsel & Bligh [5]) and the further developed 4 subscale model (McFadyen [23]) of the RIPLS. Alpha values between 0.7 and 0.8 are considered to be satisfactory [32]. Corrected item-total correlations were calculated for the four subscale model to identify items that did not correlate appropriately with the subscale score of the RIPLS. Item-total correlations between 0.4 and 0.7 are considered to be appropriate [33]. Statistical analysis was done with IBM SPSS 20. (IBM Corp. Released 2011. IBM SPSS Statistics for Windows, Version 20.0. Armonk, NY: IBM Corp. Chicago, IL, USA).

## Results

### Sample

All 19 items of the RIPLS-D were completed by 76 students (response rate 28.5%) and 76 graduates (response rate 33.8%) of which 75 students and 71 graduates indicated socio-demographic characteristics. Responder characteristics of both samples are summarized in Table 1. More than half of the respondents in each sample were nurses, about 75% had a high school degree and 88.2% (students) respectively 84% (graduates) were female.

**Table 1:** Descriptive characteristics of graduates and students who filled in the RIPLS

**Characteristics**	**Graduates**	**Students**
***Healthcare profession***	***n = 71***	***n = 75***
Nurses	43 (60.6%)	44 (58.7%)
Therapists	19 (26.8%)	14 (18.7%)
Medical laboratory/radiology assistants	5 (7.0%)	11 (14.7%)
Health Care Assistants	4 (5.6%)	6 (8.0%)
***School education***	***n = 75***	***n = 68***
Higher school degree (>10 years)	59 (78.7%)	50 (73.6%)
***Gender***	***n = 75***	***n = 68***
Female	63 (84.0%)	60 (88.2%)
***Age***	***n = 76***	***n = 68***
< 20 years old	0 (0.00%)	16 (23.5%)
20 to 25 years old	46 (60.5%)	42 (61.8%)
25 to 30 years old	22 (28.9%)	6 (8.8%)
> 30 years old	8 (10.5%)	4 (5.9%)

### Item characteristics

Item means and standard deviations are displayed in (Additional file 1: Table S1). Corrected item-total correlations showed acceptable values for all scales in the student sample. In the graduate sample, the “teamwork and collaboration” and the “positive professional identity” scales showed acceptable values. All corrected item-total correlations in the “negative professional identity” and “roles and responsibilities” scales ranged from 0.24 – 0.34.

### Internal consistency

Table 2 displays internal consistency of the RIPLS-D for both samples and the original sample with the 3-factor model. The “teamwork and collaboration” and “professional identity” scale showed good internal consistency in both samples and similar values to the English original version. The subscale “professional identity” showed a lower value for the graduate sample (Cronbach’s α = 0.42) than for the students sample (Cronbach’s α = 0.65), however a higher value compared with the original sample in Parsell and Bligh (Cronbach’s α = 0.32).

**Table 2:** Internal consistency of RIPLS-D in the two samples and in the original study

	**Graduates (n = 76)**	**Students (n = 76)**	**Parsell & Bligh (n = 120)**
Subscale 1: Teamwork and Collaboration	0.81	0.88	0.88
Subscale 2: Professional identity	0.74	0.61	0.63
Subscale 3: Roles and Responsibilities	0.42	0.65	0.32
Overall scale	0.83	0.83	0.90

Table 3 displays internal consistency of the RIPLS-D for both samples with the proposed four factor subscale model compared with data of McFadyen [23].

**Table 3:** **Internal consistency of RIPLS-D in the two samples with the four factor subscale model in comparison with data of McFadyen**[23]

	**Graduates (n = 76)**	**Students (n = 76)**	**McFadyen [**[23]**] (Data 2003; n = 348)**	**McFadyen [**[23]**] (Data 2004; n = 284)**
Subscale 1: Teamwork and Collaboration	0.81	0.88	0.79	0.88
Subscale 2: Negative Professional identity	0.46	0.78	0.60	0.76
Subscale 3: Positive Professional identity	0.76	0.82	0.76	0.81
Subscale 4: Roles and Responsibilities	0.42	0.65	0.40	0.43
Overall scale	0.83	0.83	0.84	0.89

## Discussion

This study describes the translation process of the Readiness for Interprofessional Learning Scale into German and reports on preliminary psychometric testing for two German samples of health care professionals (students and graduates). RIPLS-D showed good internal consistency in the overall scale in both sample groups. Nevertheless, the graduate sample showed unacceptable values in the “negative professional identity” and the “roles and responsibilities” sub-scales with Cronbach’s α < 0.7 [32].

The samples of McFadyen et al. [23] also showed unacceptable values in the internal consistency of the “roles and responsibilities” subscales in both undergraduate cohorts irrespective of their exposure to interprofessional learning activities. McFadyen et al. [23] argue that the roles and responsibilities sub-scale values are weak in early undergraduate students due to their lack of professional experience. Our research however found internal consistency in this scale was weaker in graduates than in students, which does not support McFadyen’s argument.

Further research into this is clearly needed to explain such differences. Various factors could be influencing these results: the cohorts exposure to various health professionals during training (students) or in their professional workplace (graduates); students and graduates may have different backgrounds regarding teamwork and we do not know at which stage a student or graduate considers himself as being part of an interprofessional team for the first time. Nevertheless, internal consistency issues were identified by McFaden and in this study, which suggests that the RIPLS “roles and responsibilities” sub-scale is unreliable.

Other translations of the RIPLS analysing psychometric properties have also shown reliability in the overall instrument but revealed problems in the factor structure. Lauffs et al. concluded that only the “teamwork and collaboration” scale was reliable and suggested further refinement of the model [24]. A following study with the same Swedish version [34] concluded a one-factor solution labelling it “Team-Player” within a larger sample.

Hayashi’s Japanese translation of a modified 15-item RIPLS [35] as used by Curran et al. [36] showed high internal consistency (α = 0.87), however, it had a different factor solution to that previously reported by Parsell and Bligh [5] and McFadyen [23]. Curran and Hayashi refer to the Readiness for Interprofessional Learning Scale in their article yet rename the instrument as “Attitudes towards Interprofessional Education” and give no rationale for the modifications made. It therefore seems possible that their instrument has a different underlying factor structure, not enabling comparisons with other RIPLS results. Another Japanese version using the 19-item RIPLS showed good values for Cronbach’s alpha in a three factor model labelled teamwork and collaboration (α = 0.92), interprofessional education opportunities (α = 0.90) and uniqueness of profession (α = 0.60) [25].

The translation into Serbian only reported Cronbach’s alpha for the whole scale revealing an adequate value of 0.84. Testing of factor structure was not reported [27]. Psychometrics of the French Canadian version have not been reported [26].

Mattick and Bligh state that future work is required regarding the development of the RIPLS and observe that modifications to the scale as well as studies using the original and modified scales do not report the same structure as the original RIPLS [37]. An extended 29-item scale has emerged a three factor sub-scale structure (teamwork and collaboration, professional identity, patient-centredness) [21,22] which also needs further verification [37]. Due to scale modification and relabeling of subscales it does not seem clear if these subscales represent “readiness for interprofessional learning” or a different concept. Williams used probabilistic test theory and performed a Rasch analysis of the RIPLS deriving a four-factor model with 17 of the original 19 items and recommending refinement and further testing [38].

The variations and translations of the instrument with various psychometrics make it difficult to compare, classify and appraise results. Ownership and copyright on instruments may be seen as counterproductive limiting freedom of research. On the other hand these arrangements help synthesize results and support instrument refinement and development. Copyright of the RIPLS would therefore support interested researchers in instrument application and enable critical appraisal of their results.

The RIPLS was developed in 1998, being one of the first instruments for measurement of attitudes toward interprofessional learning (IPL). The conceptual framework “readiness for interprofessional learning” was conceptualized by Parsell and Bligh through a range of theories, identifying key dimensions from the literature as well as by expert panels and personal experiences. Since then, researchers have not gone to the trouble of conceptualizing the framework once again or modifying and adapting the RIPLS, possibly assuming the concept to be valid. Furthermore, in the past 15 years the interprofessional education movement has been gaining momentum and conceptualization of frameworks and development of other instruments has taken place [39]. For example, the more recently developed instrument, the University of the West of England Interprofessional Questionnaire, which has four scales each measuring an individual concept; Teamwork and Communication, Interprofessional Learning, Interprofessional Interaction, Interprofessional Relationship [40].

Reliability of the RIPLS-D cannot confirm the underlying model. Other studies have shown reliability in the instruments overall scale, yet demonstrated problems in the factor structure. For these reasons, questions still remain as to what the RIPLS-D is actually measuring, and in extension which RIPLS version measures what. It remains unclear if RIPLS is simply a different instrument for measuring attitudes toward interprofessional teamwork or attitudes towards interprofessional learning. Hollar, for example, considers RIPLS as a strong instrument for measurement of health professionals’ attitudes towards team learning [41]. On the other hand, Thannhauser states the conceptualization of the terminology and constructs to be measured need a theoretical base, instead of simply modifying and rearranging existing tools hoping they will measure something or show (positive) effects in a pre-post-study [6]. This creates a strong rationale for the argument that before we continue to apply the RIPLS we need to know more about the “bigger questions” ( [37] p.141) we are hoping to address with this instrument. Qualitative research with learners at different stages of their education and early career and educators may help explore which factors are relevant today in the field of interprofessional learning and education.

Limitations: During the translation process, due to barriers in gaining corrective feedback from the developers, all conceptual questions may not have been adequately resolved resulting in possible conceptual inequivalence between the original English version and the translated German version. The German sample did not include medical or dentistry students, showing possible differences to the original sample of health professions; however, comparable professions were represented in the sample from McFadyen [23] and the German sample. In an attempt to control for possible participation bias, in that respondents were only motivated students and graduates, a reminder was sent to participants two weeks after the first email and the survey was left online longer than announced. Being an online survey based solely on voluntary participation without an incentive, a response rate of about 30% is regarded as good [42]. Nevertheless, confirmatory factor analysis of the underlying concept could not be tested because of sample size.

## Conclusion

We found limitations in internal consistency of RIPLS-D and are therefore led to question its use to measure students’ and health professionals’ attitudes towards interprofessional learning. The underlying factor structure was not able to be confirmed. Internal consistency of the instrument was not established for health professionals at different stages of their educational/professional career. Therefore, researchers should take care when using the RIPLS-D to measure change over time. Although the internal consistency is only one psychometric dimension of an instrument, it does not confirm nor support an underlying theoretical framework, in the case of RIPLS for “readiness for interprofessional learning”. The overall scale reliability does not reflect this aspect and in particular the sub-scale “roles and responsibilities” was problematic. For these reasons, the RIPLS-D cannot be recommended for use to assess this concept.

## Competing interests

The authors declare that they have no competing interests.

## Authors’ contributions

CM conceived, designed and coordinated the study, collected data and drafted the manuscript. JR participated in the statistical analysis and interpretation of data. SK participated in design and coordination of the study and data acquisition. JS was involved in the study design and revised the manuscript critically for important intellectual content. KH participated in the study design, performed statistical analysis, interpretation of data and revised and edited the manuscript. All authors read and approved the final manuscript.

## Pre-publication history

The pre-publication history for this paper can be accessed here:

http://www.biomedcentral.com/1472-6920/14/145/prepub

## Supplementary Material

Additional file 1: Table S1 Item means with standard deviations (SD) and corrected item-total correlations for the four subscales of the RIPLS-D for both samples (graduates and students). Click here for file
