# Supplementary material for: Internal consistency of the readiness for interprofessional learning scale in German health care students and professionals
Source: BMC Med Educ. 2014 Jul 16;14:145. doi: 10.1186/1472-6920-14-145 (PMC4107476; doi:10.1186/1472-6920-14-145)
Supplement: Additional file 1: Table S1 — Item means with standard deviations (SD) and corrected item-total correlations for the four subscales of the RIPLS-D for both samples (graduates and students). [file 1472-6920-14-145-S1.docx]

Table 2: Item means with standard deviations (SD) and corrected item-total correlations for the four subscales of the RIPLS-D for both samples (graduates and students)

|  | **Graduates (n=76)** | | | **Students (n=76)** | | |
| --- | --- | --- | --- | --- | --- | --- |
| ***Teamwork and collaboration*** | Mean | SD | Corrected item-total correlation | Mean | SD | Corrected item-total correlation |
| 1. Gemeinsam mit Angehörigen anderer Gesundheitsberufe zu lernen wird dazu beitragen, dass ich mich effektiver in die Teamarbeit einbringen kann | 3.61 | .82 | 0.47 | 3.74 | 1.038 | 0.68 |
| *Learning with other students will help me become a more effective member of a health care team* |  |  |  |  |  |  |
| 2. Patienten würden letztendlich profitieren, wenn die Angehörigen der Gesundheitsberufe zusammen arbeiten würden, um die Probleme der Patienten zu lösen | 4.30 | .69 | 0.50 | 4.49 | .76 | 0.70 |
| *Patients would ultimately benefit if health care students worked together to solve patient problems* |  |  |  |  |  |  |
| 3. Gemeinsames Lernen mit Angehörigen anderer Gesundheitsberufe wird meine Fähigkeit klinische Probleme zu verstehen erhöhen | 3.87 | .88 | 0.55 | 4.05 | 1.02 | 0.64 |
| *Shared learning with other health care students will increase my ability to understand clinical problems* |  |  |  |  |  |  |
| 4. Gemeinsames Lernen mit Angehörigen anderer Gesundheitsberufe während der Ausbildung wird die Beziehungen nach Berufsabschluss verbessern | 3.76 | .98 | 0.46 | 3.95 | .96 | 0.68 |
| *Learning with health care students before qualification would improve relationships after qualification* |  |  |  |  |  |  |
| 5. Kommunikative Fertigkeiten sollten gemeinsam mit Angehörigen anderer Gesundheitsberufe erlernt werden | 3.64 | 1.00 | 0.57 | 3.71 | .96 | 0.67 |
| *Communication skills should be learned with other health care students* |  |  |  |  |  |  |
| 6. Gemeinsames Lernen wird meine Meinung über andere Gesundheitsberufe positiv beeinflussen | 3.59 | .97 | 0.63 | 3.79 | 1.00 | 0.64 |
| *Shared learning will help me to think positively about other professionals* |  |  |  |  |  |  |
| 7. Damit Kleingruppenarbeit gelingt müssen sich Angehörige der Gesundheitsberufe gegenseitig vertrauen und respektieren | 4.14 | .84 | 0.35 | 4.38 | .73 | 0.52 |
| *For small group learning to work, students need to trust and respect each other* |  |  |  |  |  |  |
| 8. Es ist unerlässlich, dass alle Angehörigen der Gesundheitsberufe Kompetenzen in der Teamarbeit entwickeln | 4.33 | .76 | 0.39 | 4.24 | .83 | 0.63 |
| *Team-working skills are essential for all health care students to learn* |  |  |  |  |  |  |
| 9. Gemeinsames Lernen wird mir dabei helfen meine eigenen Grenzen zu erkennen | 3.46 | 1.00 | 0.68 | 3.57 | 1.05 | 0.47 |
| *Shared learning will help me to understand my own limitations* |  |  |  |  |  |  |

|  | **Graduates (n=76)** | | | **Students (n=76)** | | |
| --- | --- | --- | --- | --- | --- | --- |
|  | Mean | SD | Corrected item-total correlation | Mean | SD | Corrected item-total correlation |
| ***Negative Professional identity*** |  |  |  |  |  |  |
| 10. Ich möchte meine Zeit nicht damit verschwenden. gemeinsam mit Angehörigen anderer Gesundheitsberufe zu lernen | 4.00 | .82 | 0.24 | 3.92 | 1.17 | 0.63 |
| *I don't want to waste my time learning with other health care students* |  |  |  |  |  |  |
| 11. Für die Aus-, Fort- und Weiterbildung ist es nicht notwendig. dass Angehörige der Gesundheitsberufe miteinander lernen | 3.30 | 1.13 | 0.27 | 3.38 | 1.20 | 0.66 |
| *It is not necessary for undergraduate health care students to learn together* |  |  |  |  |  |  |
| 12. Das Lösen klinischer Probleme kann nur gemeinsam mit Angehörigen des eigenen Fachgebiets erlernt werden | 3.45 | 1.06 | 0.34 | 3.29 | 1.21 | 0.57 |
| *Clinical problem-solving skills can only be learned with students from my own department.* |  |  |  |  |  |  |

|  | **Graduates (n=76)** | | | **Students (n=76)** | | |
| --- | --- | --- | --- | --- | --- | --- |
|  | Mean | SD | Corrected item-total correlation | Mean | SD | Corrected item-total correlation |
| ***Positive Professional identity*** |  |  |  |  |  |  |
| 13. Gemeinsames Lernen mit Angehörigen anderer Gesundheitsberufe wird mir helfen, besser mit Patienten und anderen Fachleuten zu kommunizieren | 3.64 | .83 | 0.53 | 3.95 | .94 | 0.66 |
| *Shared learning with other health care students will help me to communicate better with patients and other professionals*. |  |  |  |  |  |  |
| 14. Ich würde es begrüßen. mit Personen anderer Gesundheitsberufe in kleinen Projekten zusammen zu arbeiten. | 3.71 | .96 | 0.57 | 3.90 | 1.07 | 0.71 |
| *I would welcome the opportunity to work on small-group projects with other health care students.* |  |  |  |  |  |  |
| 15. Gemeinsames Lernen wird dazu beitragen, die Ursache von Patientenproblemen zu ergründen | 3.59 | .87 | 0.63 | 3.95 | .92 | 0.61 |
| *Shared learning will help to clarify the nature of patient problems* |  |  |  |  |  |  |
| 16. Gemeinsames Lernen vor Abschluss der Ausbildung wird dazu beitragen, dass ich mich besser in die Teamarbeit einbringen kann | 3.37 | 1.02 | 0.54 | 3.53 | 1.03 | 0.60 |
| *Shared learning before qualification will help me become a better team worker* |  |  |  |  |  |  |

|  | **Graduates (n=76)** | | | **Students (n=76)** | | |
| --- | --- | --- | --- | --- | --- | --- |
|  | Mean | SD | Corrected item-total correlation | Mean | SD | Corrected item-total correlation |
| ***Roles and responsibilities*** |  |  |  |  |  |  |
| 17. Die Aufgabe von Pflegenden, Therapeuten und technischen Assistenten besteht vor allem darin, Ärzte zu unterstützen | 2.39 | 1.16 | 0.23 | 2.76 | 1.29 | 0.46 |
| *The function of nurses and therapists is mainly to provide support for doctors* |  |  |  |  |  |  |
| 18. Ich bin mir nicht sicher was meine professionelle Rolle ist. | 2.20 | 1.05 | 0.31 | 2.17 | 1.18 | 0.48 |
| *I'm not sure what my professional role will be* |  |  |  |  |  |  |
| 19. Ich muss mir deutlich mehr Wissen und Fertigkeiten aneignen, als Angehörige anderer Gesundheitsberufe | 2.64 | .90 | 0.24 | 2.87 | 1.11 | 0.43 |
| *I have to acquire much more knowledge and skills than other health care students* |  |  |  |  |  |  |
